# Rhizosphere processes by the nickel hyperaccumulator *Odontarrhena chalcidica* suggest Ni mobilization

**DOI:** 10.1007/s11104-023-06161-w

**Published:** 2023-07-12

**Authors:** Sören B. L. Risse, Markus Puschenreiter, Alice Tognacchini

**Affiliations:** 1 Department of Forest and Soil Sciences, Institute of Soil Research, University of Natural Resources and Life Sciences, Konrad-Lorenz-Straße 24, 3430 Tulln, Austria; 2 Centre for Microbiology and Environmental Systems Science, Department for Environmental Geosciences, University of Vienna, Althanstrasse 14, 1090 Vienna, Austria

**Keywords:** Hyperaccumulation, Trace metals, Metal mobilization, Ultramafic soils

## Abstract

**Background and aims:**

Plant Ni uptake in aboveground biomass exceeding concentrations of 1000 μg g^−1^ in dry weight is defined as Ni hyperaccumulation. Whether hyperaccumulators are capable of mobilizing larger Ni pools than non-accumulators is still debated and rhizosphere processes are still largely unknown. The aim of this study was to investigate rhizosphere processes and possible Ni mobilization by the Ni hyperaccumulator *Odontarrhena chalcidica* and to test Ni uptake in relation to a soil Ni gradient.

**Methods:**

The Ni hyperaccumulator *O. chalcidica* was grown in a pot experiment on six soils showing a pseudo-total Ni and labile (DTPA-extractable) Ni gradient and on an additional soil showing high pseudo-total but low labile Ni. Soil pore water was sampled to monitor changes in soil solution ionome, pH, and dissolved organic carbon (DOC) along the experiment.

**Results:**

Results showed that Ni and Fe concentrations, pH as well as DOC concentrations in pore water were significantly increased by *O. chalcidica* compared to unplanted soils. A positive correlation between Ni in shoots and pseudo-total concentrations and pH in soil was observed, although plant Ni concentrations did not clearly show the same linear pattern with soil available Ni.

**Conclusions:**

This study shows a clear root-induced Ni and Fe mobilization in the rhizosphere of *O. chalcidica* and suggests a rhizosphere mechanism based on soil alkalinization and exudation of organic ligands. Furthermore, it was demonstrated that soil pH and pseudo-total Ni are better predictors of Ni plant uptake in *O. chalcidica* than labile soil Ni.

**Supplementary Information:**

The online version contains supplementary material available at 10.1007/s11104-023-06161-w.

## Introduction

Nickel hyperaccumulation in plants has primarily evolved as an adaptation to Ni-rich ultramafic soils in different world regions (Baker and Brooks 1989; Lange et al. 2017; van der Ent et al. 2016, 2018). The threshold level of nickel hyperaccumulation was set to 1000 μg g^−1^ in dry shoots (Baker and Brooks 1989; van der Ent et al. 2013). Such plants have received growing scientific attention due to their potential applications in the remediation of metal contaminated soils and more recently in nickel phytomining (Bani et al. 2015a, b; Chaney et al. 2007; Krämer 2005; Lombi et al. 2000; Nkrumah et al. 2016; Pardo et al. 2018; Rosenkranz et al. 2019; van der Ent 2015; Wenzel et al. 1999). To allow those exceptionally high metal concentrations in plant tissues, enhanced metal uptake and translocation mechanisms should have developed in hyperaccumulator plants (Baker 1981, 1987). Whether hyperaccumulators are able to modify metal availability in their soil and to access larger metal fractions which are unavailable to non-accumulator plants is still debated. Recent studies have contributed to clarify plant internal (Assunção et al. 2003; Brooks 1998; Krämer et al. 1996, 1997, 2000; Krämer 2010; Lombi et al. 2000) and rhizosphere processes (Álvarez-López et al. 2021; Dessureault-Rompré et al. 2010; Puschenreiter et al. 2005; Wenzel et al. 1999, 2003) associated with metal hyperaccumulation. Nevertheless, the mechanisms of metal acquisition and specific rhizosphere processes of hyperaccumulator plants are still largely unknown. Labile metal fractions assessed on soil often poorly correlate with metal uptake in hyperaccumulators and literature shows contradictory results. While some research provides evidence of increased Ni plant uptake with higher Ni soil availability (Centofanti et al. 2012; Massoura et al. 2004), other studies suggest the contrary (Bani et al. 2014; Noller 2017; Puschenreiter et al. 2019). Several studies support the hypothesis that hyperaccumulators rely on metal mobilization from less available metal fractions (Álvarez-López et al. 2021; Chardot-Jacques et al. 2013; Puschenreiter et al. 2005; Wenzel et al. 2003); however, literature also suggest that hyperaccumulators access the same metal pools as non-accumulators (Echevarria et al. 2006; Hammer et al. 2006; Hutchinson et al. 2000; Massoura et al. 2004; Salt et al. 2000). Non-linear relations would imply a role of active Ni mobilization processes at the roots level, which might not necessarily be targeting the hyperaccumulated metals (such as Ni) but could possibly occur to enhance plant uptake of other essential nutrients. As possible mechanism for high Ni plant accumulation, it was suggested that hyperaccumulator species may release root exudates containing Ni-chelators with the potential to enhance Ni uptake and translocation (Álvarez-López et al. 2021; Puschenreiter et al. 2005; Salt et al. 2000; Wenzel et al. 2003). Despite the extensive knowledge about rhizosphere process involved in plant nutrient acquisition, the role of root induced metal mobilization in hyperaccumulator plants has not been yet clarified. Higher dissolved organic carbon (DOC) measured in rhizosphere soil suggested an active release of roots exudates by the Ni hyperaccumulator *Noccaea goesingensis* (Wenzel et al. 2003) and *Odontarrhena serpillyfolia* (Álvarez-López et al. 2021) which was associated with higher Ni availability in rhizosphere soil. Puschenreiter et al. (2005) also found some indication for the release of citric acid in the rhizosphere of *Noccaea goesingensis*. Li et al. (2012) indicated higher organic acid levels in the rhizosphere of the hyperaccumulator *Sedum alfredii* compared with a non-hyperaccumulator ecotype, which was associated with higher Zn mineral dissolution. On the contrary, in other studies no or little indication of root-induced solubilization was observed (Salt et al. 2000; Whiting et al. 2001; Zhao et al. 2001). To the best of our knowledge, no studies have been specifically investigating rhizosphere processes of the Ni hyperaccumulator *Odontarrhena chalcidica* (formerly *Alyssum murale*).

The aim of this study was to investigate if: i) Ni accumulation in hyperaccumulator plants is following a gradient of total and available Ni in soil; ii) evidence of root exudation and Ni mobilization can be observed in the rhizosphere of a Ni hyperaccumulator; iii) co-mobilization of Ni with other nutrients such as Fe and P occurs; iv) root-induced changes in soil pH can be linked to enhanced Ni soil availability. We hypothesise that Ni plant uptake rather correlates with total soil Ni than plant available Ni fractions and that Ni (and possibly Fe and P) might be co-mobilized in the rhizosphere of hyperaccumulators due to plant root activity. To test our hypotheses, a pot experiment was conducted with the Ni hyperaccumulator *O. chalcidica* on seven soils ranging from moderate to pronounced ultramafic characteristics and showing a gradient in total and available Ni.

## Material and methods

### Experimental soil characterisation

Experimental soils were collected from an ultramafic forest area near Redlschlag (eastern Austria) previously described by Puschenreiter et al. (2005), Wenzel and Jockwer (1999) and Wenzel et al. (2003). Soils were sieved < 4 mm in the field and left to air-drying for several weeks. To investigate effects of increasing Ni concentrations, a linear Ni gradient was artificially created by mixing two soils in different percentages, resulting in four mixed soils. Therefore, a soil with lower Ni concentrations (S1) was mixed, based on the dry weight, with a higher Ni soil (S6) as shown in Table 1. An additional soil, representing a subsoil exposed to the surface by a landslide (LS), was included in the experiment because it was characterised by high pseudo-total Ni but low labile Ni content. All soil analyses were performed in triplicates on < 2 mm sieved, air-dried subsamples and batch extractions were corrected for the total water content determined at 105 °C. Pseudo-total element concentrations were determined by microwave (CEM Mars 6) assisted digestion of 0.5 g milled subsamples in HNO_3_ 65% and HCl 37% at 1:3 ratio (*aqua regia*) for 40 min at 200 °C according to Austrian standards (ÖNORM L 1085,2009). Readily-available metal fraction was assessed by 0.01 M Sr(NO_3_)_2_ extraction (Everhart et al. 2006) as adapted by Madden (1988) using 1:4 *w/v* ratio and 2 h shaking. Complexable metals were determined through DTPA (diethylene triamine pentaacetic acid) extraction after Lindsay and Norvell (1978) with 1:2 *w/v* and 2 h shaking. Plant available P was extracted according to Olsen (1954) as described in Schoenau and O'Halloran (2008) and P determined by molybdenum blue colometry (Murphy and Riley 1962). Cation exchange capacity (CEC) was estimated as the sum of the exchangeable cations Ca, Mg and K using 0.1 M BaCl_2_ extraction according to Austrian standards (ÖNORM L^1086^-^89^,^1989^). Soil pH was measured in ultrapure H_2_O (conductivity: 0.055 < 0.080 mS) at 1:2.5 *w/v* with a pH meter (SCHOTT ProLab 4000). Trace elements in soil digests, Sr(NO_3_)_2_ and DTPA extraction were determined with an ICP-MS (NexION 2000, Perkin Elmer). Macronutrients for *aqua regia* and CEC assays with an ICP-OES (Optima 8300, Perkin Elmer).

**Table 1:** Soil mixing scheme

Soil	% of S1	% of S6
S1	100	0
S2	80	20
S3	60	40
S4	40	60
S5	20	80
S6	0	100
LS	–	–

### Pot experiment and pore water sampling

In order to observe changes in Ni hyperaccumulation and rhizosphere processes of *Odontarrhena chalcidica* at increasing soil Ni concentrations, a pot experiment with a soil Ni gradient was set up and plants were grown under controlled conditions. For S1 to S6, 450 g soil and for LS 550 g soil, because of higher density, was added to PVC pots in four experimental replicates. Experimental control consisted of unplanted pots, which were also setup for each soil in four replicates. Rhizon pore water (PW) samplers (Rhizosphere Research Products, Wageningen, NL) were installed in each pot to allow soil pore water sampling. *Odontarrhena chalcidica* seeds were germinated in washed vermiculite and transplanted after seven days into the pot experiment. After seven more days, when the plants showed 2–3 pair of leaves, one plant was kept per pot. Plants were grown in a growth cabinet (CLF Plant Climatics, SE59-AR2cLED), with the following settings: 16 h photoperiod with 400 μmol photons m^−2^ s^−1^, temperatures of 24/18 °C during the light/dark period and humidity maintained at 60%. The pots were arranged randomly and shuffled every two days, soil moisture was kept gravimetrically at 60% water holding capacity (WHC) by daily watering with ultrapure H_2_O. Soil pore water was sampled four times during the experiment: seven days after transplantation (DAT) of seedlings into the pots (T0), 21 DAT (T1), 49 DAT (T2), and 71 DAT (T3), when the experiment ended. Each time 10 mL of filtered (mean pore size of rhizon sampler = 0.15 μm) PW was sampled from water saturated pots and analysed for pH (SCHOTT ProLab 4000 pH meter), P content (Murphy and Riley 1962), dissolved organic carbon (DOC) (Vario TOC Cube, Elementar) and trace metals (ICP-MS, NexION 2000, Perkin Elmer).

### Plant biomass analyses

Plant shoots were harvested after 71 DAT, carefully rinsed with deionised water, oven dried at 60 °C for 38 h, weighed and milled. Shoots were digested in a 4:1 mixture of HNO_3_ 65% and H_2_O_2_ 30% in a microwave oven (CEM Mars 6) at 200 °C for 25 min. Elemental concentrations in plant biomass were analysed for trace elements with ICP-MS (NexION 2000, Perkin Elmer) and for macronutrients with ICP-OES (Optima 8300, Perkin Elmer).

### Soil analyses after the pot experiment

To investigate changes to soil parameters resulting from the pot experiment, Sr(NO_3_)_2_, DTPA, pH and Olsen-P analyses were performed on planted and control pots as previously described. The seven initial soils were also included in all analyses for comparing with the initial conditions without operational biases.

### Statistical analyses

Differences in physiochemical parameters of pore water, plant and soil samples between planted replicates were assessed using two-way ANOVA with Tukey’s HSD post-hoc test. If normal distribution was not met data was either log-transformed or non-parametric Kruskal–Wallis H test was applied, followed by Wilcoxon rank sum test. Differences between planted and unplanted replicates within each soil group were assessed using Student’s t-test. If normal distribution was not met, data were either log-transformed or a non-parametric Mann–Whitney-U test applied. To test for correlation between two variables Pearson’s product-momentum correlation was performed after checking for normality and homoscedasticity of the data. All statistical analyses were carried out using R version 4.2.2 (2022–10-31). Sample size was n = 4 for pore water, plant, and soil samples after the pot experiment and n = 3 for initial soil samples and a significance level of *p* < 0.05 was used.

## Results

### Experimental soil characterisation

An overview of the main characteristics of the experimental soils, such as pseudo-total elemental concentrations, DTPA- and Sr(NO_3_)_2_-extractable metals, plant available P, CEC and pH is shown in Table 2. *Aqua regia* digestion as well as DTPA and Sr(NO_3_)_2_ extraction confirmed that soil mixing successfully created a Ni gradient from S1 to S6 in both labile and pseudo-total concentrations. Pseudo-total Ni concentrations ranged from 552 mg kg^−1^ (S1) to 1465 mg kg^−1^ (S6) and was even higher in LS (1613 mg kg^−1^). DTPA-extractable Ni accounted for about 10% of pseudo-total concentrations and increased threefold from S1 (41.6 mg kg^−1^) to S6 (158 mg kg^−1^) as well, whereas LS showed low DTPA-extractable Ni (53.1 mg kg^−1^, ~ 3% of pseudo-total Ni). Sr(NO_3_)_2_-extractable Ni was distinctly low in LS (376 µg kg^−1^) and increased twofold from S1 (641 µg kg^−1^) to S6 (1247 µg kg^−1^). Regarding nutrient contents, pseudo-total K (2.23 g kg^−1^) concentrations decreased to 1.03 g kg^−1^ K from S1 to S6, while P, S and Fe showed the opposite trend with lowest concentrations in S1 and highest in S6. LS showed comparable concentration regimes regarding Fe, Mg, K and Na within the S1 to S6 range but was considerably higher in S (732 mg kg^−1^) and lower in pseudo-total P (131 mg kg^−1^). All soils of the experiment showed very low concentrations of plant-available P, especially low in LS (1.17 mg kg^−1^) and marginally increasing from S1 to S6 (2.94 to 6.51 mg kg^−1^). Soil pH ranged from slightly acidic in S1 (5.93) towards nearly neutral (6.46) in S6 and was alkaline in LS (8.08).

**Table 2:** Pseudo-total, DTPA- and Sr(NO_3_)_2_-extractable elemental concentrations, Olsen-P, CEC and pH of the experimental soil characterisation before the pot experiment, presented as mean ± standard deviation (*n* = 3)

	S1	S2	S3	S4	S5	S6	LS
	g kg ^−1^	g kg ^−1^	g kg ^−1^	g kg ^−1^	g kg ^−1^	g kg ^−1^	g kg ^−1^
Pseudo-total							
Fe	56.3 ± 4.7	57.7 ± 3.9	60.1 ± 3.7	60.4 ± 6.3	66.5 ± 1.4	69.1 ± 1.8	59.3 ± 3.4
Mg	74.1 ± 5.7	87.4 ± 2.3	110 ± 16	117 ± 5.8	136 ± 4.4	151 ± 2.3	136 ± 4.1
Ca	4.18 ± 0.4	3.82 ± 0.4	3.79 ± 0.6	3.41 ± 0.5	2.33 ± 0.1	1.66 ± 0.2	5.36 ± 0.5
K	2.23 ± 0.1	2.28 ± 0.3	2.04 ± 0.4	1.95 ± 0.2	1.32 ± 0.04	1.03 ± 0.1	2.06 ± 0.6
Cr	1.14 ± 0.1	1.21 ± 0.1	1.50 ± 0.3	1.52 ± 0.1	1.98 ± 0.2	2.04 ± 0.1	1.33 ± 0.9
Mn	1.08 ± 0.3	1.08 ± 0.6	1.12 ± 0.4	1.15 ± 0.1	1.30 ± 0.6	1.33 ± 0.4	1.10 ± 0.6
	mg kg ^−1^	mg kg ^−1^	mg kg ^−1^	mg kg ^−1^	mg kg ^−1^	mg kg ^−1^	mg kg ^−1^
Ni	552 ± 52	715 ± 75	857 ± 60	1026 ± 74	1249 ± 46	1465 ± 58	1613 ± 8.3
Co	72.4 ± 1.8	75.9 ± 3.8	80.8 ± 0.8	88.4 ± 5.0	106 ± 13	113 ± 4.1	107 ± 2.9
Cu	13.1 ± 1.5	16.7 ± 3.9	14.9 ± 0.4	18.1 ± 2.1	19.2 ± 0.9	21.7 ± 2.4	40.6 ± 2.4
Zn	62.7 ± 1.5	63.4 ± 8.6	65.0 ± 5.3	60.3 ± 0.9	70.6 ± 8.3	59.4 ± 1.4	53.4 ± 5.5
P	235 ± 14	252 ± 9.9	257 ± 35	259 ± 12	281 ± 10	297 ± 20	131 ± 6.8
S	334 ± 3.5	370 ± 35	369 ± 58	390 ± 11	372 ± 19	414 ± 34	732 ± 53
Na	167 ± 16	166 ± 30	163 ± 47	125 ± 31	51.8 ± 5.2	21.4 ± 11	64.5 ± 47
DTPA							
Ni	41.6 ± 0.5	61.8 ± 0.7	84.9 ± 2.4	107 ± 1.4	137 ± 7.2	158 ± 7.1	53.1 ± 0.6
Fe	317 ± 1.9	300 ± 2.8	275 ± 4.0	255 ± 1.3	250 ± 5.9	212 ± 7.9	17.5 ± 0.7
Cr	< 0.06	< 0.06	< 0.06	< 0.06	< 0.06	< 0.06	< 0.06
Co	3.20 ± 0.05	2.19 ± 0.2	2.75 ± 0.1	1.91 ± 0.03	1.88 ± 0.02	1.78 ± 0.1	0.57 ± 0.04
Cu	0.87 ± 0.1	1.09 ± 0.2	1.23 ± 0.03	1.52 ± 0.02	2.70 ± 1.3	2.08 ± 0.1	1.61 ± 0.02
Zn	4.02 ± 0.2	3.48 ± 0.4	3.07 ± 0.1	2.77 ± 0.1	2.69 ± 0.1	2.21 ± 0.3	0.30 ± 0.1
Olsen-P	2.94 ± 0.1	3.53 ± 0.4	3.59 ± 0.2	4.21 ± 0.5	6.20 ± 0.2	6.51 ± 0.9	1.17 ± 0.2
	µg kg ^−1^	µg kg ^−1^	µg kg ^−1^	µg kg ^−1^	µg kg ^−1^	µg kg ^−1^	µg kg ^−1^
Sr(NO_3_)_2_							
Ni	641 ± 1.8	809 ± 15	920 ± 10	1033 ± 14	1157 ± 19	1247 ± 9.0	376 ± 14
Fe	52.4 ± 4.7	56.8 ± 9.2	77.4 ± 20	63.3 ± 32	108 ± 94	117 ± 104	43.8 ± 28
Cr	10.8 ± 0.7	10.2 ± 0.4	13.6 ± 5.0	11.5 ± 2.5	12.3 ± 0.4	< 1.22	< 1.22
Co	84.7 ± 2.7	40.8 ± 3.1	36.8 ± 0.5	18.6 ± 0.2	17.3 ± 0.9	12.7 ± 0.5	4.31 ± 0.2
Cu	17.0 ± 6.5	10.6 ± 3.3	9.84 ± 2.5	68.5 ± 95	15.8 ± 3.4	8.36 ± 17	4.45 ± 0.6
Zn	135 ± 1.3	103 ± 1.2	87 ± 7.6	106 ± 66	61 ± 5.7	210 ± 1.4	33.8 ± 0.8
	cmol_c_ kg^−1^	cmol_c_ kg^−1^	cmol_c_ kg^−1^	cmol_c_ kg^−1^	cmol_c_ kg^−1^	cmol_c_ kg^−1^	cmol_c_ kg^−1^
CEC	18.8 ± 0.2	20.0 ± 0.6	21.4 ± 0.5	22.5 ± 0.4	24.3 ± 1.3	27.5 ± 0.8	14.1 ± 0.1
	[-]	[-]	[-]	[-]	[-]	[-]	[-]
pH	5.93 ± 0.03	6.11 ± 0.06	6.18 ± 0.01	6.25 ± 0.02	6.33 ± 0.01	6.48 ± 0.08	8.08 ± 0.03

### Shoot biomass and elemental concentrations

**Fig. 1:**
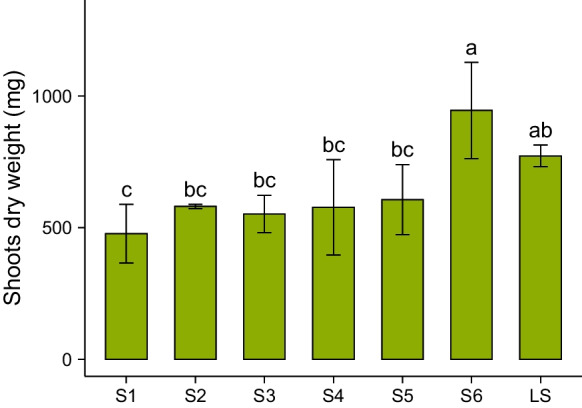
Shoot dry weight of *Odontarrhena chalcidica* per soil, presented as mean ± standard deviation (*n* = 4). Statistical differences (*p* < 0.05) among different soils are indicated with letters

Average values of plant dry weight (DW) are presented in Fig. 1 and Table 2. *Odontarrhena chalcidica* grown on S1 showed the lowest biomass (477 mg DW), whereas S6 plants grew nearly twice as much (946 mg DW), significantly higher than S1 to S5 plants and in the same range as LS plants (773 mg DW). S1 to S5 did not show any differences in DW of the shoots and only S1 plants had significantly lower biomass than LS plants.

**Fig. 2:**
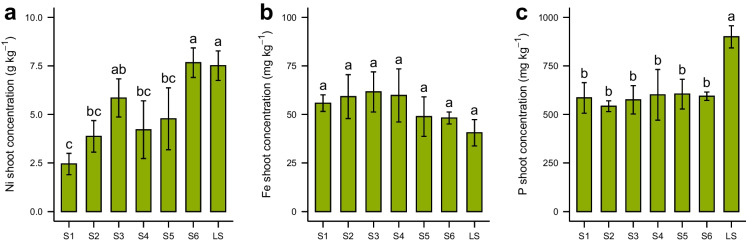
Dry shoot concentrations of Ni (**a**), Fe (**b**) and P (**c**) of *Odontarrhena chalcidica* growing on the different soils, presented as mean ± standard deviation (*n* = 4). Statistical differences (*p* < 0.05) among soils is indicated with letters

Elemental concentrations in shoots are shown in Table 3. The lowest dry shoots Ni concentrations were measured in S1 and S2 plants (2.44 and 3.87 g kg^−1^) and the highest in S6 and LS plants (7.67 and 7.51 g kg^−1^, Fig. 2 a). S4 and S5 showed plant Ni concentrations comparable with S1, S2 and S3 without significant differences. No significant differences were observed among Fe concentrations in plant shoots (41 to 62 mg kg^−1^) from all soils (Fig. 2 b), while *O. chalcidica* showed significantly elevated shoot P concentrations (900 mg kg^−1^) when growing on LS soil compared to the other soils (Fig. 2 c).

**Table 3:** Shoot biomass and total concentrations of Ni, Fe, P and other elements of *Odontarrhena chalcidica*, presented as mean ± standard deviation (*n* = 4), letters indicate ANOVA and Tukey’s HSD results (*p* < 0.05)

	S1	S2	S3	S4	S5	S6	LS
	mg DW	mg DW	mg DW	mg DW	mg DW	mg DW	mg DW
Shoot biomass	477 ± 112 **c**	581 ± 8.5 **bc**	552 ± 72 **bc**	577 ± 181 **bc**	606 ± 133 **bc**	946 ± 183 **a**	773 ± 41 **ab**
	g kg ^−1^	g kg ^−1^	g kg ^−1^	g kg ^−1^	g kg ^−1^	g kg ^−1^	g kg ^−1^
Ni	2.44 ± 0.5 **c**	3.87 ± 0.8 **bc**	5.79 ± 1.1 **ab**	4.08 ± 1.7 **bc**	4.78 ± 1.6 **bc**	7.67 ± 0.8 **a**	7.51 ± 0.8 **a**
K	9.37 ± 2.0 **a**	8.06 ± 1.3 **a**	8.53 ± 2.0 **a**	8.22 ± 2.7 **a**	6.76 ± 2.6 **a**	5.99 ± 0.7 **a**	8.91 ± 0.7 **a**
Mg	5.24 ± 0.5 **ab**	5.49 ± 0.9 **ab**	6.00 ± 0.7 **ab**	6.51 ± 0.6 **a**	5.48 ± 0.8 **ab**	4.68 ± 1.0 **b**	2.88 ± 0.6 **c**
Ca	9.02 ± 1.5 **b**	8.27 ± 1.3 **b**	7.82 ± 1.8 **b**	8.58 ± 1.1 **b**	8.43 ± 2.1 **b**	7.15 ± 1.5 **b**	14.7 ± 5.2 **a**
S	1.40 ± 1.6 **b**	1.22 ± 0.3 **b**	1.25 ± 0.2 **b**	1.27 ± 0.2 **b**	1.21 ± 0.3 **b**	0.93 ± 0.7 **b**	2.51 ± 0.4 **a**
	mg kg ^−1^	mg kg ^−1^	mg kg ^−1^	mg kg ^−1^	mg kg ^−1^	mg kg ^−1^	mg kg ^−1^
Fe	56 ± 4.3 **a**	59 ± 11 **a**	62 ± 10 **a**	60 ± 14 **a**	49 ± 10 **a**	48 ± 3.1 **a**	41 ± 6.8 **a**
P	570 ± 89 **b**	542 ± 28 **b**	601 ± 150 **b**	609 ± 122 **b**	605 ± 77 **b**	594 ± 3.3 **b**	900 ± 58 **b**
Zn	152 ± 62 **a**	142 ± 28 **ab**	126 ± 16 **abc**	78 ± 17 **bc**	81 ± 23 **bc**	102 ± 15 **abc**	62 ± 10 **c**
Mn	116 ± 20 **a**	92 ± 20 **ab**	61 ± 17 **bc**	45 ± 4.0 **c**	35 ± 10 **c**	42 ± 7.4 **c**	45 ± 10 **c**

### Pore water sampling

**Fig. 3:**
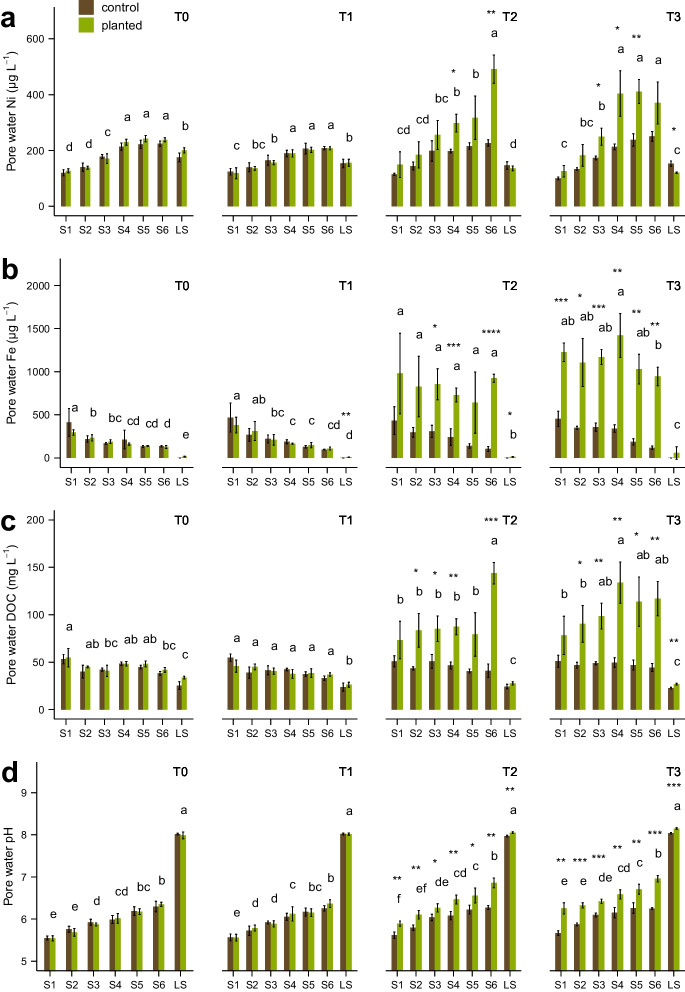
Pore water concentrations of Ni (**a**), Fe (**b**), DOC (**c**) and pH (**d**) of pots with *Odontarrhena chalcidica* and controls for the samplings T0 to T3, presented as mean ± standard deviation (*n* = 4). Letters indicate significance between planted soils (*p* < 0.05), asterisks show significance between planted and control pots (*p* < 0.05 = *, *p* < 0.01 = **, *p* < 0.001 = ***)

Pore water samples were collected to detect root-induced changes to soil solution in the rhizosphere and results are shown in Fig. 3. At the beginning of the experiment (T0) DOC concentrations in PW were highest in planted S1 (54.7 µg L^−1^) and lowest in LS control pots (25.3 µg L^−1^), but no major differences between the pots were evident. Control pots remained comparable for DOC concentrations from T0 to T3, whereas increasing DOC concentrations were measured in planted pots after 49 days (T2), significant for S2, S3, S4 and especially S6 (144 µg L^−1^, S6 control: 41 µg L^−1^). At T3, planted S5 and LS pots also showed significantly elevated DOC concentrations in PW and planted S4 contained highest DOC contents in PW (134 µg L^−1^). The pH as well as Fe and Ni concentrations in PW between planted and control pots likewise showed an increase starting at T2 and continuing for T3. In this regard alkalinization of the pH of the PW at T3 was clearly visible for S6 (pH increase of 0.71) and least pronounced but still significant for LS (pH increase of 0.11). Nickel concentrations in PW resulted highest for S6 at T2 (265 µg L^−1^) with more than two-fold increase compared to controls. At T3 Ni concentrations significantly increased for S3, S4 and S5, with 76, 191 and 174 µg L^−1^, respectively, corresponding to a 44%, 90% and 73% increase compared to the unplanted controls. In contrast, for LS there was a small reduction of Ni concentrations, which started at T2 and became significant at T3 (33 µg L^−1^). Iron concentrations in soil PW showed the most pronounced increase along the experiment with elevated concentrations at T2 significant for S3 and strongly significant for S4 and S6. At T3 dissolved Fe concentrations in planted pots were highest in S4 (1420 µg L^−1^), S6 increased more than 8 times (828 µg L^−1^) compared to controls, whereas LS was the only soil without a significant increase with very low values (29 µg L^−1^) in general. Phosphorus concentrations in PW were very low, so that the majority of the samples were below the quantification limit of 20 µg L^−1^ and thus could not be reliably determined.

### Changes in soil characteristics after the pot experiment

**Fig. 4:**
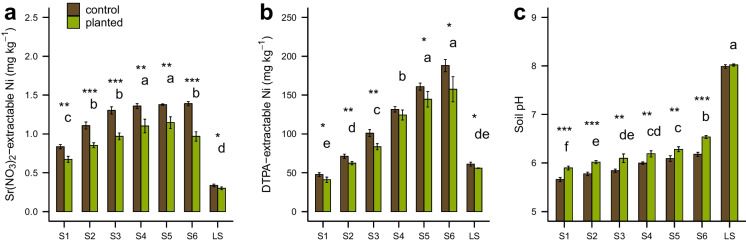
Sr(NO_3_)_2_-extractable Ni (**a**), DTPA-extractable Ni (**b**) and pH (**c**) as well as pH (**d**) of the different soils after the pot experiment, presented as mean ± standard deviation (*n* = 4). Letters indicate significance between planted soils (*p* < 0.05), asterisks show significance between planted and control pots (*p* < 0.05 = *, *p* < 0.01 = **, *p* < 0.001 = ***)

Soil pH, Olsen-P and extractable Ni (DTPA and Sr(NO_3_)_2_ extraction) are shown in Table 4 and Fig. 4. Planted pots for both assessments showed a significant reduction of labile Ni compared to control pots for almost all soils. The reduction was higher in Sr(NO_3_)_2_-extractable Ni ranging from 38 µg kg^−1^ (11.3%) in LS up to 422 µg kg^−1^ (30.4%) in S6 and less pronounced in DTPA-extractable Ni with lowest values in S1 and LS, 6.8 and 5.2 mg kg^−1^ (14.2% and 8.5%) and highest in S6, 30.5 mg kg^−1^ (16.2%). No significant differences between planted and control pots were detected for DTPA- or Sr(NO_3_)_2_-extractable Fe. A significant alkalinization of soil pH of soils S1 to S6 after the pot experiment was measured with averaged values ranging from 0.19 (S4 and S5) to 0.34 (S6). In contrast, no significant differences in soil pH between planted and control pots were evident for LS. Plant-available P did not significantly change before and after the pot experiment and also no differences between planted and control pots were visible.

**Table 4:** DTPA- and Sr(NO_3_)_2_-extractable elemental concentrations, Olsen-P and pH of the experimental soils after the pot experiment of planted and control pots, presented as mean ± standard deviation (*n* = 4). Letters indicate significance between planted soils (*p* < 0.05), asterisks show significance between planted and control pots (*p* < 0.05 = *, *p* < 0.01 = **, *p* < 0.001 = ***)

	S1		S2		S3		S4		S5		S6		LS	
	planted	control	planted	control	planted	control	planted	control	planted	control	planted	control	planted	control
	mg kg ^−1^		mg kg ^−1^		mg kg ^−1^		mg kg ^−1^		mg kg ^−1^		mg kg ^−1^		mg kg ^−1^	
DTPA														
Ni	40.9 ± 3.6 **e**	47.7 ± 2.4*	62.4 ± 2.1 **d**	71.1 ± 2.8**	83.6 ± 4.1 **c**	101 ± 4.5**	124 ± 6.4 **b**	132 ± 3.5	145 ± 10.1 **a**	161 ± 4.5*	157 ± 16 **a**	188 ± 7.9*	55.9 ± 0.4 **de**	61.1 ± 2.5*
Fe	419 ± 49 **ab**	387 ± 18	397 ± 23 **ab**	363 ± 40	364 ± 62 **ab**	363 ± 32	434 ± 20 **a**	404 ± 23	379 ± 16 **ab**	377 ± 8.2	352 ± 14 **b**	337 ± 15	46.8 ± 3.1 **c**	40.9 ± 4.5
Mn	21.8 ± 2.3 **c**	22.9 ± 1.5	24.2 ± 1.4 **bc**	23.9 ± 3.2	23.6 ± 4.4 **bc**	27.7 ± 4.6	28.2 ± 1.4 **ab**	28.0 ± 0.4	27.5 ± 1.0 **ab**	28.0 ± 1.5	29.4 ± 1.8 **a**	28.9 ± 1.0	8.12 ± 0.2 **d**	7.30 ± 0.8
Co	1.58 ± 0.2 **b**	1.72 ± 0.1	1.82 ± 0.1 **b**	1.82 ± 0.3	1.87 ± 0.4 **b**	2.18 ± 0.4	2.37 ± 0.1 **a**	2.35 ± 0.1	2.30 ± 0.1 **a**	2.35 ± 0.1	2.41 ± 0.2 **a**	2.45 ± 0.1	0.65 ± 0.01 **c**	0.59 ± 0.1
Cu	1.32 ± 0.2 **e**	1.17 ± 0.2	1.48 ± 0.1 **de**	1.42 ± 0.1	1.68 ± 0.2 **d**	1.76 ± 0.2	2.40 ± 0.1 **bc**	2.28 ± 0.2	2.67 ± 0.1 **b**	2.62 ± 0.1	3.00 ± 0.1 **a**	2.92 ± 0.1	2.27 ± 0.1 **c**	2.22 ± 0.1
Zn	3.20 ± 0.3 **a**	3.37 ± 0.2	2.64 ± 0.1 **b**	2.65 ± 0.2	2.27 ± 0.3 **bc**	2.37 ± 0.2	2.33 ± 0.3 **b**	2.18 ± 0.1	1.85 ± 0.2 cd	2.09 ± 0.4	1.78 ± 0.1 **d**	1.95 ± 0.1	0.33 ± 0.1 **e**	0.38 ± 0.2
Olsen-P	7.84 ± 1.5 **a**	8.05 ± 2.0	7.68 ± 1.3 **a**	7.59 ± 2.2	6.97 ± 1.0 **a**	7.97 ± 0.3	7.43 ± 0.6 **a**	6.73 ± 0.5	6.43 ± 1.1 **a**	7.60 ± 0.6	6.82 ± 0.6 **a**	7.08 ± 0.4	1.81 ± 0.2 **b**	2.60 ± 0.2*
Sr(NO_3_)_2_														
Mn	1.33 ± 0.4 **ab**	1.48 ± 0.5	1.38 ± 0.2 **ab**	1.38 ± 0.6	0.92 ± 0.5 **ab**	1.51 ± 0.6	1.08 ± 0.5 **ab**	1.29 ± 0.9*	0.91 ± 0.1 **ab**	1.03 ± 0.5*	0.74 ± 0.3 **b**	0.87 ± 0.3**	0.07 ± 0.02 **c**	0.06 ± 0.01
	µg kg ^−1^		µg kg ^−1^		µg kg ^−1^		µg kg ^−1^		µg kg ^−1^		µg kg ^−1^		µg kg ^−1^	
Ni	672 ± 40 **c**	835 ± 26**	853 ± 32 **b**	1107 ± 44***	968 ± 45 **b**	1302 ± 47***	1102 ± 87 **a**	1358 ± 32**	1144 ± 75 **a**	1377 ± 11**	967 ± 59 **b**	1389 ± 27***	299 ± 18 **d**	337 ± 17*
Fe	109 ± 28 **a**	76.8 ± 29	96.1 ± 8.3 **a**	72.3 ± 22	94.6 ± 40 **a**	77.3 ± 20	127 ± 13 **a**	127 ± 18	118 ± 14 **a**	103 ± 8.6	132 ± 55 **a**	118 ± 29	54.0 ± 52 **a**	42.3 ± 43
Co	43.8 ± 9.8 **a**	49.1 ± 12	42.9 ± 6.2 **a**	45.0 ± 17	29.5 ± 13 **ab**	46.5 ± 15	32.8 ± 2.2 **ab**	38.3 ± 2.6*	27.0 ± 0.7 **b**	30.6 ± 1.1*	20.8 ± 1.3 **b**	24.8 ± 0.8*	4.09 ± 1.4 **c**	3.10 ± 0.3
Cu	22.3 ± 0.6 **a**	27.0 ± 8.8	19.8 ± 5.7 **a**	18.8 ± 1.3	16.7 ± 4.3 **ab**	14.8 ± 4.9	10.8 ± 2.1 **b**	15.2 ± 4.3	11.5 ± 1.4 **b**	18.0 ± 3.7	12.1 ± 3.3 **b**	12.5 ± 3.7	< 1.16	< 1.16
Zn	109 ± 3.5 **a**	120 ± 4.2*	88.9 ± 9.4 **ab**	100 ± 17	69.9 ± 6.2 **bc**	74.9 ± 3.4	54.9 ± 2.9 **cde**	62.9 ± 7.7	64.3 ± 26 **bcd**	56.5 ± 5.9	44.3 ± 1.0 **de**	49.9 ± 4.0	37.5 ± 3.5 **e**	42.3 ± 4.7
	[-]		[-]		[-]		[-]		[-]		[-]		[-]	
pH	5.90 ± 0.04 **f**	5.66 ± 0.04***	6.02 ± 0.03 **e**	5.78 ± 0.03***	6.10 ± 0.1 **de**	5.84 ± 0.04**	6.19 ± 0.1 cd	6.00 ± 0.02**	6.28 ± 0.1 **c**	6.09 ± 0.1**	6.54 ± 0.03 **b**	6.18 ± 0.04***	8.02 ± 0.02 **a**	7.99 ± 0.04

## Discussion

### Plant biomass and shoot analyses

The study of plant growth and Ni accumulation in typical to weakly ultramafic soils provided interesting insights into plant growth and the acquisition of Ni and other nutrients by *O. chalcidica*. Remarkably, the highest plant shoots biomass was obtained from the two soils with the more pronounced ultramafic characteristics (soils S6 and LS, Fig. 1), despite the very different plant availability of essential nutrients such as P, N (Fig. S2, supplementary information) and Fe (Table 2). Similarly, the highest Ni shoot uptake in *O. chalcidica* was obtained from the more distinctly ultramafic soils S6 and LS (Fig. 2), suggesting that specific ultramafic soil properties might promote Ni shoot uptake more than Ni availability itself. Ni accumulation in shoots from all soils was above the hyperaccumulation threshold of 1000 μg g^−1^ in dry shoots (Baker and Brooks 1989; van der Ent et al. 2013) and comparable with previous experimental work on *O. chalcidica* (Bani et al. 2015a, b; Rosenkranz et al. 2019; Tognacchini et al. 2020). Of particular note is the significantly higher P uptake by plants in LS (Fig. 2c), which has the lowest P availability (Table 2). This might indicate a very efficient P uptake strategy or favourable soil conditions for P uptake in LS soil, such as higher pH or very low concentrations of soluble Fe in pore water.

### Soil pore water analyses

The significant increase in Ni pore water (PW) concentrations in planted pots from T0 to T3 in soils S3, S4, S5 and S6, and marginally S1 and S2 (Fig. 3) is a clear evidence of plant-induced Ni solubilization from insoluble Ni pools to soil water solution. Conversely, the decrease in PW Ni concentrations in soil LS over the course of the experiment indicates either that no Ni mobilization occurred, or that plant uptake had a stronger effect than solubilization. The strong Fe solubilization observed in the planted pots during the experiment in all soils except LS (Fig. 3) further confirms rhizosphere processes involved in metal mobilization; the high correlation (*r* = 0.81) between Ni and Fe solubilization also suggests a co-mobilization of those metals from the same soil fractions, possibly from Ni associated with soil Fe oxides, which has been shown to be one of the main sources of labile Ni in ultramafic soils (Álvarez-López et al. 2021; Chardot et al. 2005; Massoura et al. 2004). As for Ni, also the limited Fe solubilization in LS is reflecting the different geochemistry and rhizosphere processes for this soil. Furthermore, the significant increase in DOC in planted pots along the experiment (Fig. 3) is likely due to root-related increase of soluble organic compounds and a possible indication of root exudation by *O. chalcidica* in all soils. The increased DOC in pore water also seems to promote Ni and Fe soil solubilization in planted pots, as suggested by the high positive correlation between ΔDOC /ΔNi and ΔDOC/ΔFe in PW at T3 (*r* = 0.96 and *r* = 0.86 respectively, Fig. S1 supplementary information). As further indication that plant-derived DOC is involved in Ni and Fe solubilization, no Ni and Fe PW mobilization was observed in soil LS, where DOC increase was negligible. Similarly to our results, an increased Ni solubility in rhizosphere of ultramafic soils compared to bulk soil was observed in Wenzel et al. (2003) and Álvarez-López et al. (2021) on, respectively, the Ni hyperaccumulator species *Noccaea goesingensis* and *Odontarrhena serpyllifolia*. In both studies, increased Ni solubility was associated with higher DOC and pH in soil and a positive correlation between soil Ni availability and DOC in the rhizosphere was also observed. Puschenreiter et al. (2005) reported higher concentrations of oxalic and citric acid in the rhizosphere compared to bulk soil of *Thlaspi goesingense* (syn. *Noccaea goesingensis*) from a natural ultramafic site. Based on chemical speciation analysis (MINTEQA2), Wenzel et al. (2003) suggested the formation of Ni-organic complexes in the rhizosphere of *N. goesingensis* and that organic ligands excreted by roots form stronger complexes with Ni than organic compounds from bulk soil. This might explain the enhanced Ni present in PW, which seems to be in complexed form with organic ligands from root exudation.

The pH increase of pore water samples from rooted pots along the experiment (Fig. 3) suggests a significant root-induced soil alkalinization, as observed in previous studies on hyperaccumulator plants (Álvarez-López et al. 2021; Kukier et al. 2004; Luo et al. 2000; Puschenreiter et al. 2005; Singer et al. 2007; Wenzel et al. 2003, 2004). Wenzel et al. (2003) hypothesised that the pH increase in the rhizosphere of *N. goesingensis* could be related with the release of hydroxyl ions during mineral dissolution of Mg and Ni-bearing orthosilicates. From a hydroponic test with *O. chalcidica* (Tognacchini et al. 2022, unpublished) a substantial pH increase in a sampling solution was also observed within two hours of root exposure, which implies other mechanisms of pH increase besides mineral dissolution. As previously proposed in literature, possible beneficial effect of pH increase in the rhizosphere might be the stabilization of metal–organic ligands (Li et al. 2003; van der Ent et al. 2016), thus keeping in solution metals as Ni and Fe associated with soluble organic compounds. Because of the very low P concentrations in PW (results not shown) and partially unclear results, a thorough discussion about P geochemistry and mobilization is unfortunately limited. Despite this, a general trend in P_PW_ increase in planted pots was observed, which would suggest a plant-promoted solubilization. Exception, again, is made for soil LS, where a significant P depletion occurred, which might be reflected in the enhanced plant uptake observed in plant shoots of *O. chalcidica.*

### Soil Ni availability and plant uptake

Another main research question we wanted to investigate was whether a linear relation between a soil Ni gradient and plant uptake could be observed. Previous studies conducted on ultramafic soils from the same location show a weak correlation of Ni concentration in *Noccaea goesingensis* shoots with soil available Ni assessed by Sr(NO_3_)_2_ extraction, DTPA extraction and DGT (diffusive gradients in thin films) assessment (Noller 2017; Puschenreiter et al. 2019). Bani et al. (2014) observed that Ni accumulation in *Odontarrhena chalcidica* was independent from soil DTPA-extractable Ni. On the contrary, Centofanti et al. (2012) showed that Ni accumulation in *Alyssum corsicum* was dependent upon the solubility of the Ni mineral present in the growth substrate. From our results, the increasing Ni availability from soil S1 to soil S6 (Ni_PW_, Ni_DTPA_ and Ni_Sr(NO3)2_) was not entirely reflected in shoot Ni uptake in *O. chalcidica* and seems to be limited to a specific range of Ni availability (e.g. from soil S1 to S3). This shows limitations in predicting shoot Ni uptake based on soil availability assessments such as Ni_PW_, Ni_DTPA_, Ni_Sr(NO3)2_ and could partially explain contradictory literature results. Surprisingly, our experiment revealed that Ni uptake by shoots of *O. chalcidica* is highly predictable from the soil pseudo-total Ni and pH of soil and PW, (respectively: *r* = 0.87, *r* = 0.72 and r = 0.75; see Fig. S1 in supplementary information), suggesting that soil total Ni pools and pH might be better predictors of shoot Ni concentrations than the Ni plant-available fractions. This might indicate that: i) Ni mobilization processes from non-available Ni pools might play a central role in hyperaccumulation, or/and that ii) Ni uptake might be regulated by soil pH. As already underlined in literature, soil pH seems to have a central role in plant Ni uptake (Everhart et al. 2006; Ghafoori et al. 2022; Li et al. 2003; Kukier et al. 2004). In several studies a higher Ni accumulation by *Alyssum* (synonymous *Odontarrhena*) species was observed as soil pH was raised and thus soil available Ni (Ni_DTPA_, Ni_Sr(NO3)2_ and Ni biosensor) declined (Everhart et al. 2006; Kukier et al. 2004; Li et al. 2003). Since increasing soil ultramafic properties (and total Ni) was associated with increasing pH (Table 2), the apparent linear relation of shoot uptake with total soil Ni may actually be a bias related to the effect of soil pH. Another relevant aspect is the opposite response to soil alkalinization of extraction-based Ni availability assessments (DTPA and Sr(NO_3_)_2_) compared to Ni in PW. The reduction in DTPA and Sr(NO_3_)_2_ extractable Ni after plant growth (Fig. 4) cannot be justified by plant uptake or PW removal alone, which accounts for approximately half of the Ni loss and it is seemingly a combined effect of immobilization due to pH increase. Considering the soil extractable Ni fractions only (Ni_DTPA_ and Ni_Sr(NO3)2_; Fig. 4) we would have concluded that rhizosphere processes have caused Ni immobilization, while PW analyses resulted to be crucial in observing Ni solubilization. In contrast with our results, Álvarez-López et al. (2021) measured a significantly higher available Ni (Sr(NO_3_)_2_ and DGT) in rhizosphere soil of *O. serpillifoilia* compared to bulk soil, showing that rhizosphere processes in ultramafic soil induced Ni mobilization. Being a field study, in Álvarez-López et al. (2021) the long-term rhizosphere effect might justify the different results obtained compared to our pot experiment. Contrasting literature results can be found regarding the capability of hyperaccumulator plants to access larger Ni pools than non-accumulators. For example, it was shown that hyperaccumulators within the genus *Odontarrhena* accesses the same Ni pool as non-hyperaccumulators (Massoura et al. 2004; Shallari et al. 2001). In contrast, Chardot-Jacques et al. (2013) observed enhanced dissolution of a Ni-bearing mineral in the rhizosphere of the Ni hyperaccumulator *Bornmuellera emarginata* (syn. *Leptoplax emarginata*). Although evidence of active Ni solubilization was observed in the rhizosphere of *O. chalcidica* in the present experiment, it cannot be stated whether this indicates mobilization from larger Ni pools that are not available to non-hyperaccumulators. The linearity observed between soil Ni total pools and plant uptake, would suggest that *O. chalcidica* could access non-available pools, while Ni uptake in *O. chalcidica* seems to derive from Ni solubilized from adsorbed and surface complexed soil Ni fractions which are also potentially available to non-accumulators. A clear link between Ni solubilization and Ni shoot uptake was not observed and high Ni accumulation in *O. chalcidica* occurred in soil LS without signs of solubilization. This suggests that even when root mobilization occurs, it is not a strategy to enhance Ni plant uptake. As already suggested by Álvarez-López et al. (2021), Ni mobilization appears to be a consequence of rhizosphere processes targeting other nutrients.

## Conclusions

This study presents new insights into rhizosphere processes involved in Ni biogeochemistry of the Ni hyperaccumulator species *O. chalcidica*. We demonstrated that Ni uptake by plants was favoured by ultramafic soil characteristics and correlated well with soil characteristics like pseudo-total Ni content or pH, whereas Ni bioavailability assessments (Ni_DPTA_ and Ni_Sr(NO3)2_) resulted to be poor predictors of plant Ni uptake. A strong increase in pore water Ni and Fe concentrations combined with elevated DOC content and alkalinization in most soils suggested solubilization of Ni and Fe mediated by exudation of organic ligands. This led us to conclude that root-related activities clearly influence the fate of Ni in the rhizosphere*.* However, it remains unclear whether *O. chalcidica* has access to a larger Ni pool that is unavailable to non-hyperaccumulator plants. Further studies should investigate in more detail the composition of root exudates from *O. chalcidica* and include comparisons with non-accumulator plant species adapted to ultramafic soils.

### Supplementary Information

Below is the link to the electronic supplementary material.

Supplementary file1 (PDF 32 KB)

Supplementary file2 (PDF 8 KB)

## Data Availability

The datasets generated during and/or analysed during the current study are available from the corresponding author on reasonable request.
