# Supplementary figures and images for: Rhizosphere processes by the nickel hyperaccumulator Odontarrhena chalcidica suggest Ni mobilization
Source: Plant Soil. 2023 Jul 12;495(1-2):43–56. doi: 10.1007/s11104-023-06161-w (PMC10834574; doi:10.1007/s11104-023-06161-w)

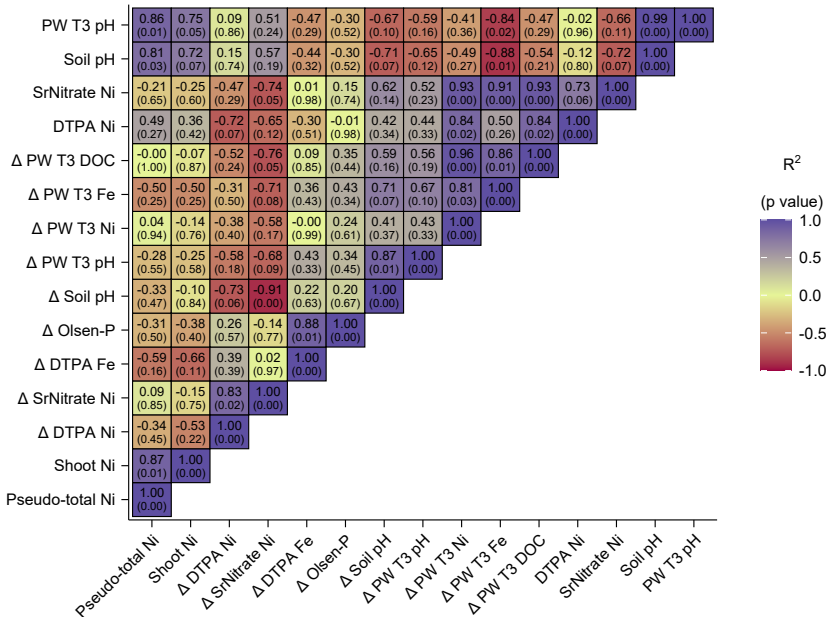

Supplement: Supplementary file 1 — Supplementary file1 (PDF 32 KB) [file 11104_2023_6161_MOESM1_ESM.pdf]

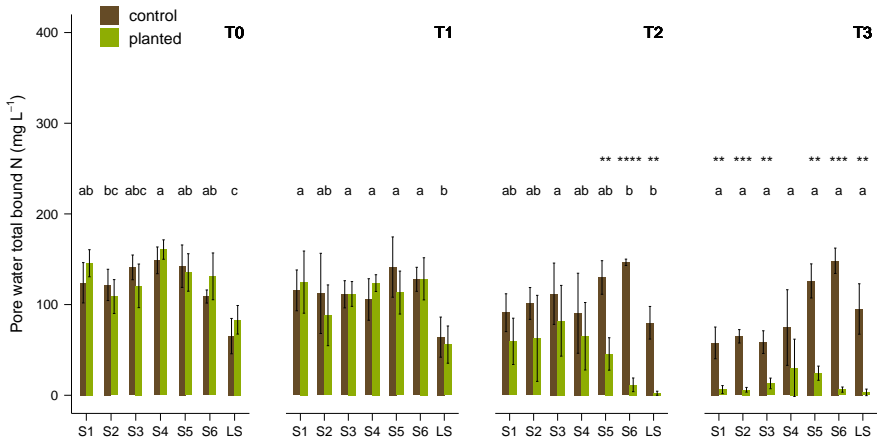

Supplement: Supplementary file 2 — Supplementary file2 (PDF 8 KB) [file 11104_2023_6161_MOESM2_ESM.pdf]
